# Assessing the Utility of Large Language Models in Guiding Dental Practitioners on Pediatric Patient Care: A Comparative AI Study

**DOI:** 10.4317/jced.63136

**Published:** 2025-09-01

**Authors:** Mithula Raj, Vignesh Ravindran, Abirami Arthanari

**Affiliations:** 1 Post graduate, Dept of Pediatric and Preventive Dentistry, Saveetha Dental College & Hospitals, Saveetha Institute of Medical and Technical Sciences, Saveetha University, Chennai, Tamil Nadu, India; 2 Associate Professor, Dept of Pediatric and Preventive Dentistry, Saveetha Dental College & Hospitals, Saveetha Institute of Medical and Technical Sciences, Saveetha University, Chennai, Tamil Nadu, India; 3 Lecturer, Dept of Forensic Odontology, Saveetha Dental College & Hospitals, Saveetha Institute of Medical and Technical Sciences, Saveetha University, Chennai, Tamil Nadu, India

## Abstract

**Background:**

Large Language Models (LLMs) are transforming clinical decision-making by offering rapid, context-aware access to evidence-based knowledge. However, their efficacy in pediatric dentistry remains underexplored, especially across multiple LLM platforms.

Objective: To comparatively evaluate the clinical quality, readability, and originality of responses generated by nine contemporary LLMs for pediatric dental queries.

**Material and Methods:**

A cross-sectional study assessed the performance of ChatGPT-3.5, ChatGPT-4o, Gemini 2.0, Gemini 2.5, Claude 3.5 Haiku, Claude 3.7 Sonnet, Grok-3, Grok-3 Mini, and DeepSeek-V3. Twenty pediatric dental questions were posed in one-shot queries to each LLM. Responses were evaluated by ten pediatric dental experts using the Modified Global Quality Scale (MGQS), Flesch Reading Ease Score (FRES), Flesch-Kincaid Grade Level (FKGL), and Turnitin Similarity Index. ANOVA and Cohen’s Kappa were used for statistical analysis.

**Results:**

ChatGPT-4o demonstrated the highest overall MGQS (4.28 ± 0.24), followed by ChatGPT-3.5 (3.45 ± 0.27). DeepSeek-V3 scored lowest (2.18 ± 0.19). Topic-wise, ChatGPT-4o consistently outperformed others across all subdomains. FRES and FKGL scores indicated moderate readability, with Claude models exhibiting the highest linguistic complexity. Turnitin analysis revealed low-to-moderate similarity across models. Inter-rater agreement was substantial (κ = 0.78).

**Conclusions:**

Among evaluated LLMs, ChatGPT-4o exhibited superior performance in clinical relevance, coherence, and originality, suggesting its potential utility as an adjunct in pediatric dental decision-making. Nonetheless, variability across models underscores the need for critical appraisal and cautious integration into clinical workflows.

** Key words:**Artificial Intelligence, Clinical decision support, Health Communication, Large language models, Natural Language Processing.

## Introduction

The integration of artificial intelligence (AI) into clinical healthcare has initiated a significant transformation in how medical knowledge is accessed, interpreted, and applied by practitioners. Among the most impactful innovations in this domain are large language models (LLMs)—transformer-based generative systems capable of understanding and generating human-like language with contextual fluency and probabilistic reasoning [1,2]. These models hold considerable promise for enhancing healthcare delivery by enabling the rapid synthesis of evidence-based information, especially in specialties requiring nuanced decision-making such as pediatric dentistry [3].

Since the launch of OpenAI’s ChatGPT in late 2022, LLMs have shown utility in streamlining clinical documentation, supporting continuing education, and improving real-time communication within healthcare settings [4,5]. Building on this foundation, several advanced models have since emerged, each introducing unique design features and optimization strategies. For instance, Anthropic’s Claude incorporates ethical guardrails and improved interpretability to support safer medical discourse. Google DeepMind’s Gemini employs multimodal reasoning and fact-grounding to enhance diagnostic precision. Grok, developed by xAI, features live web integration to improve temporal relevance, while DeepSeek emphasizes retrieval-augmented generation to provide accurate responses with reduced latency.

In pediatric dentistry, early detection and management of caries, trauma, developmental anomalies, and behavioral concerns are critical to ensuring favorable long-term outcomes. In such contexts, LLMs may serve as valuable adjuncts for clinical decision-making. Pediatric dentists often face time-sensitive decisions involving preventive care, sedation protocols, fluoride application, and age-specific treatment planning. Access to accurate, contextually relevant, and easily interpreTable guidance can significantly impact the quality of care delivered [6]. While traditional resources such as clinical guidelines, textbooks, and expert consultation remain essential, LLMs offer a supplementary avenue for exploring differential diagnoses, procedural workflows, and emerging literature.

Nonetheless, integrating LLMs into clinical dental practice poses several challenges. Despite their linguistic capabilities, these models can demonstrate variability in domain-specific accuracy, are prone to factual errors or hallucinations, and may occasionally diverge from established clinical protocols [7]. Additionally, most current evaluations of LLMs in healthcare focus on single-model performance—primarily ChatGPT—and tend to emphasize patient or caregiver communication rather than clinician-oriented use cases [8]. This highlights a significant gap in understanding how various LLMs perform when delivering targeted, clinically relevant information to dental professionals.

As AI-based tools become more embedded within digital health ecosystems, there is a growing need for comprehensive evaluation frameworks that go beyond surface-level assessments of language coherence. Critical parameters such as clinical accuracy, contextual appropriateness, readability, originality, and linguistic complexity must be rigorously examined to determine a model’s reliability as a clinical support tool [9]. Furthermore, clinicians must remain mindful of the limitations of these systems. LLMs lack the experiential insight, ethical reasoning, and diagnostic accountability intrinsic to human expertise [10]. Their outputs should be viewed as informational aids, not replacements for clinical judgment, and must be corroborated with validated evidence and professional oversight.

Given these considerations, the present study adopts a structured, multidimensional approach to evaluate the performance of ChatGPT, Claude, Gemini, Grok, and DeepSeek, focusing on their ability to provide clinically relevant, evidence-based guidance for dental practitioners managing pediatric patients.

Material And Methods

- Study Design and Ethical Considerations

This study employed a cross-sectional prospective design to compare the clinical relevance, response quality, and readability of generative large language models (LLMs) in answering pediatric dental queries. The study adhered to the principles outlined in the Declaration of Helsinki (2013 revision). As it involved only publicly available, non-identifiable data and no human or animal participants, formal ethical approval was not required. However, the protocol received methodological clearance from the Institutional Review Board (IRB) of the affiliated institution.

- Selection of LLMs and Question Development

A total of nine contemporary generative LLMs were selected for evaluation, encompassing diverse AI architectures and vendors. These included:

1. ChatGPT-3.5,

2. ChatGPT-4o,

3. DeepSeek-V3,

4. Gemini 2.0,

5. Gemini 2.5,

6. Grok-3,

7. Grok-3 Mini,

8. Claude 3.5 Haiku, and

9. Claude 3.7 Sonnet.

An overview of these models is provided in  Table 1.

Twenty clinical questions were developed by two board-certified general dental practitioners, each with over ten years of experience. The questions were reflective of real-world challenges commonly encountered in pediatric dentistry. Topics spanned caries management, dental trauma, developmental anomalies, oral habits, and preventive care. All questions were open-ended to elicit comprehensive, descriptive responses rather than binary or limited outputs. Difficulty was not stratified, enhancing ecological validity by simulating authentic clinical scenarios.

- Standardized Query Protocol

To replicate real-world usage, each LLM was queried once per question by a single author. No iterative rephrasing, clarification, or follow-up prompting was permitted. This one-shot querying model was adopted to assess each model’s inherent ability to generate informative, contextually appropriate responses—an essential feature in clinical decision-making.

All LLMs were accessed on March 25, 2025, via Google Search using a newly created user account. Browser cookies and cache were cleared before each session to eliminate algorithmic bias. For each model-question pair, a separate chat session was initiated to avoid residual contextual influence from previous prompts. Responses were recorded in their original, unaltered form, without the use of prompt engineering, to reflect a typical end-user experience.

- Response Compilation and Quality Evaluation

All responses were transcribed verbatim into a master document for evaluation. A panel of ten experienced pediatric dental specialists—selected via publicly accessible institutional directories—assessed the responses. Each evaluator had substantial academic and clinical expertise. They were trained remotely in the use of the Modified Global Quality Scale (MGQS), which was adapted from previous literature on LLM assessment in healthcare communication [11].

The MGQS employed a 5-point Likert scale, where:

● Score 1 indicated responses of deficient quality with minimal informational value,

● Score 5 represented exemplary answers with comprehensive, coherent, and evidence-aligned content ( Table 2).

The modal score assigned to each model per question—reflecting the most frequently occurring rating—was designated as the representative score for that entry. Emphasis during scoring was placed on the completeness of content, accuracy, clinical applicability, and linguistic fluency.

Readability Assessment

To evaluate linguistic accessibility, the responses were subjected to readability analysis using the Flesch Reading Ease Score (FRES) and Flesch-Kincaid Grade Level (FKGL) metrics. These indices were computed as follows:

● FRES = 206.835 – 1.015 × (total words ÷ total sentences) – 84.6 × (total syllables ÷ total words)

● FKGL = 0.39 × (total words ÷ total sentences) + 11.8 × (total syllables ÷ total words) – 15.59

Interpretation of FRES:

● 90–100: Easily understood by elementary students

● 60–70: Suitable for high school readers

● 30–50: Appropriate for college-level comprehension

● <30: Reserved for professional or specialized texts

FKGL values ranged from 0 to 18, indicating escalating complexity. Scores:

● 0–6: Basic literacy

● 7–12: Intermediate to high school level

● 13–18: Advanced, collegiate level

Microsoft guidelines recommend a FRES of 60–70 and FKGL of 7–8 for general-purpose documents. These metrics enabled a comparative assessment of how intelligible the outputs were to the general dental community.

Originality and Plagiarism Analysis

The originality of LLM outputs was analyzed using Turnitin® (www.turnitin.com), a widely adopted plagiarism detection system. Each LLM response was independently uploaded, and Similarity Index Scores were computed. The levels of content duplication were categorized as:

● 0%: Fully original

● 1–24%: Low similarity

● 25–49%: Moderate similarity

● 50–74%: High similarity

● 75–100%: Extensive overlap with existing sources

- Statistical Analysis

Data were analyzed using IBM SPSS Statistics Version 22.0 (IBM Corp., Chicago, IL, USA). Descriptive statistics—including means, medians, and standard deviations—were used to summarize scores across MGQS, FRES, and FKGL metrics. Normality was tested using the Shapiro–Wilk test, which guided the use of appropriate inferential tests.

To identify statistically significant differences between models, a one-way Analysis of Variance (ANOVA) was conducted for MGQS, FRES, and FKGL scores. A *p-value* of <0.05 was considered statistically significant. Additionally, Cohen’s Kappa (κ) coefficient was used to evaluate inter-rater reliability among expert scorers.

## Results

- Demographic Characteristics of Evaluating Pediatric Dentists

A total of ten pediatric dental professionals participated as evaluators in the assessment of responses generated by the large language models (LLMs). The evaluative cohort demonstrated equal gender representation, with 5 males (50%) and 5 females (50%). Regarding occupational designation, none of the participants identified exclusively as clinicians. Precisely half of the respondents (n = 5; 50%) were full-time academicians, while the remaining 50% (n = 5) maintained dual roles as both academicians and clinicians.

The mean chronological age of the participants was 33.8 ± 2.04 years, indicating a relatively youthful yet professionally mature demographic. The mean duration of professional experience in pediatric dentistry was 8.7 ± 1.49 years, whereas prior exposure to artificial intelligence applications in clinical or academic contexts remained limited, with a mean AI experience of 0.8 ± 0.42 years ( Table 3).

The inter-rater reliability analysis performed using Kappa statistics, yielded a kappa value of 0.78, indicating a substantial level of agreement between the evaluators

Comparative Evaluation of MGQS Scores Across LLMs

The Modified Global Quality Scale (MGQS) was employed to quantitatively assess the perceived quality of answers generated by each LLM. Statistically significant intergroup differences were observed (*p* < 0.001).

ChatGPT-4o achieved the highest MGQS score (4.28 ± 0.24), demonstrating superior consistency and reliability in response quality. ChatGPT-3.5 followed with a mean score of 3.45 ± 0.27, reflecting moderately high performance. Conversely, DeepSeek-V3 registered the lowest mean score (2.18 ± 0.19), signifying comparatively suboptimal response quality. Other models, including Gemini 2.5 (3.33 ± 0.30), Gemini 2.0 (3.18 ± 0.28), Grok-3 (3.02 ± 0.19), Grok-3 mini (2.79 ± 0.16), Claude 3.5 Haiku (2.77 ± 0.20), and Claude 3.7 Sonnet (2.60 ± 0.24) exhibited intermediate performance ( Table 4).

- Topic-Specific Variation in MGQS Performance

A more granular evaluation of MGQS scores, stratified by thematic domain, revealed that ChatGPT-4o consistently demonstrated exceptional performance across all pediatric dentistry subfields. For questions related to growth and development, ChatGPT-4o obtained a mean MGQS score of 4.25, followed closely by ChatGPT-3.5 (3.45). In the trauma-related domain, ChatGPT-4o exhibited peak performance (4.32), substantially outperforming other models including Claude 3.7 Sonnet (2.43) and DeepSeek-V3 (2.12).

In the dental caries subset, ChatGPT-4o again achieved the highest mean score (4.23), while DeepSeek-V3 recorded a mean of 2.17. For topics involving oral hygiene, ChatGPT-4o scored 4.18, with ChatGPT-3.5 performing well at 3.56, whereas Grok-3 mini and Claude 3.7 Sonnet scored below 3.0. Within the habit intervention and pharmacological guidance domains, ChatGPT-4o again outperformed others with mean scores of 4.35 and 4.45, respectively. In contrast, Claude 3.7 Sonnet and DeepSeek-V3 performed relatively poorly across these domains. All thematic comparisons yielded highly significant results (*p* < 0.001) ( Table 5).

- Readability Assessment: Flesch Reading Ease

The Flesch Reading Ease scores reflected the accessibility of responses generated by the LLMs. Although Claude 3.5 Haiku produced the most readable content (70.72 ± 4.06), this did not necessarily translate into higher content quality as per MGQS. ChatGPT variants—ChatGPT-4o (66.24 ± 6.95) and ChatGPT-3.5 (65.65 ± 5.14)—also maintained readability within the “standard” range. Gemini 2.0 and Claude 3.7 Sonnet exhibited lower readability scores (62.42 ± 7.40 and 62.90 ± 3.16, respectively), approaching the threshold of more complex textual presentation. Statistically significant differences were observed among all models (*p* = 0.001) ( Table 6).

- Textual Complexity: Flesch-Kincaid Grade Level

The Flesch-Kincaid Grade Level metric was used to determine the educational level required to comprehend the generated responses. The range of mean grade levels varied notably, from 6.82 ± 0.90 in Claude 3.5 Haiku (suggestive of higher accessibility) to 15.25 ± 22.92 in Gemini 2.0, indicating considerable textual inconsistency and potential overcomplexity. ChatGPT models maintained relatively balanced scores: ChatGPT-4o (9.40 ± 2.48) and ChatGPT-3.5 (9.10 ± 1.60). The observed differences were statistically significant (*p* = 0.044), suggesting varying degrees of linguistic sophistication among LLMs ( Table 7).

- Plagiarism Analysis: Similarity Index Scores

The similarity index, calculated using Turnitin, evaluated the originality of the AI-generated content. Claude 3.5 Haiku (1%), Grok-3 mini (2%), and ChatGPT-4o (3%) demonstrated the lowest similarity indices, reflecting high levels of textual uniqueness. Grok-3 exhibited the highest similarity score (13%), followed by Gemini 2.0 (10%) and Claude 3.7 Sonnet (9%). Despite apparent trends in content originality, the inter-model comparison did not reach statistical significance (*p* = 0.34) ( Table 8).

## Discussion

This study aimed to systematically evaluate the performance of nine state-of-the-art generative large language models (LLMs) in responding to pediatric dentistry queries posed by parents. The evaluation used a multidimensional framework incorporating the Modified Global Quality Scale (MGQS), Flesch readability indices, and textual originality through similarity scoring. Among the models assessed, ChatGPT-4o consistently outperformed the others in both quality and thematic consistency, achieving the highest MGQS scores across all subdomains. In contrast, DeepSeek-V3 and Claude 3.7 Sonnet showed relatively poor performance. The rationale for employing a multidimensional comparison lay in the need to assess AI-generated content not only for readability or originality but also for clinical relevance, coherence, and domain specificity—criteria often underrepresented in AI evaluation studies.

The use of ten experienced pediatric dental professionals, with balanced gender representation and a mean of nine years of clinical experience, ensured that expert assessments were grounded in real-world clinical perspectives. A high inter-rater reliability (κ = 0.78) validated the robustness of the subjective scoring, reinforcing the decision to prioritize expert evaluations over general user perceptions. This approach addressed a limitation noted in prior work by Nahir (2025), which emphasized readability and general quality but lacked evaluation by domain experts [11].

Unlike earlier studies [12], which typically focused on a single model or a narrow set of tasks, the present research covered a broader spectrum of LLMs and included domain-specific variations across six core pediatric dentistry themes. While Nahir (2025) observed higher quality scores for parent-facing questions, our findings show that ChatGPT-4o consistently maintained high MGQS scores across both general and technical queries, reflecting strong contextual adaptability [11]. The poor performance of DeepSeek-V3 may stem from limitations in its training data, particularly with regard to pediatric dental content, or inadequate fine-tuning on relevant medical corpora—factors that likely contributed to vague or inconsistent outputs.

In terms of readability, Claude 3.5 Haiku achieved the highest Flesch Reading Ease scores, partially aligning with Nahir’s earlier observations [13]. However, these higher readability scores did not correlate with higher MGQS ratings, indicating a disconnect between linguistic simplicity and the informativeness of the content. ChatGPT-4o stood out for maintaining a balance between clarity and complexity. It produced responses that were both readable and content-rich, a dual achievement that remains rare in previous evaluations. This suggests a maturation in hybrid transformer architectures that are capable of delivering human-aligned utility.

The Flesch-Kincaid Grade Level analysis further supported the idea that overly simplistic text may compromise content depth. Gemini 2.0 showed the highest variability in grade levels, likely due to inconsistent sentence structuring and lexical usage. Conversely, the ChatGPT models exhibited consistent linguistic performance, with grade levels falling within the collegiate range. This makes them particularly suitable for educated caregivers or early-career dental professionals seeking clinical guidance [14].

Originality analysis, based on similarity indices, revealed an encouraging trend: most LLMs demonstrated low textual overlap. Notably, Claude 3.5 Haiku and ChatGPT-4o yielded the lowest similarity scores. Although these differences were not statistically significant, the findings suggest that newer LLMs are increasingly capable of producing contextually rephrased content rather than relying on verbatim reproduction [15]. This has important implications for their ethical use in academic, advisory, and clinical settings where originality is a prerequisite.

The present study stands apart from previous investigations through its comprehensive analytic framework, combining quality evaluation via MGQS, readability scoring, and similarity indexing. Unlike prior studies that assessed individual models or specific medical fields, this research provides a broader comparative lens—particularly timely as AI tools become more embedded in clinical decision-making, including in general dentistry.

A notable strength of this study lies in its triangulated approach, which integrates evaluations of linguistic complexity, content fidelity, and originality. Earlier studies have evaluated ChatGPT’s performance within dental contexts [16], but none have offered a comparative assessment across modern LLMs or addressed thematic variability across subdomains such as dental trauma, oral hygiene, and pharmacologic protocols. By including multiple models, this study offers general practitioners evidence on selecting the most reliable LLM for time-sensitive clinical queries [17].

The clinical implications are significant. ChatGPT-4o demonstrated the highest overall performance, delivering responses that were contextually appropriate, lexically coherent, and consistently high in quality across all domains. These characteristics support its use as a supplementary tool for clinical decision-making and academic reference [18]. Its high Flesch Reading Ease Score (66.24) and low similarity index (3%) further suggest its applicability in clinical environments, where content must be both understandable and ethically sound. In contrast, models such as DeepSeek-V3 and Claude 3.7 Sonnet displayed inconsistent performance, indicating that they may not yet be suitable for clinical deployment without further refinement.

Despite these strengths, several limitations must be acknowledged. First, the LLM responses were based on fixed prompts crafted by the research team. No iterative refinement or advanced prompt engineering was used, which could otherwise enhance response specificity. Second, the models’ knowledge cutoffs (e.g., ChatGPT-3.5’s training ending in June 2024) limited their ability to address questions requiring the latest clinical guidelines or emerging evidence [19]. Third, the exclusive use of English restricts the applicability of findings across multilingual contexts. Additionally, while the question set covered key pediatric dentistry themes, it did not extend to other areas such as general dentistry, geriatric care, syndromic diagnoses, or telehealth integration—an area ripe for future exploration.

From an academic lens, the study highlighted persistent challenges in AI-generated synthesis of nuanced, evidence-based recommendations—particularly for case-specific or layered clinical scenarios. For example, questions involving decoronation in trauma cases exposed limitations in ChatGPT-4o and ChatGPT-3.5, which, while effective in delivering general information, lacked depth in responses requiring higher-order reasoning or citation-backed justification. These findings align with Dermata (2025), who noted the shortcomings of current generative models in handling open-ended, clinically complex queries [12].

Looking forward, our results support the development of evidence-linked LLMs with real-time access to clinical databases. Such systems could provide dynamic referencing and greater contextual accuracy in clinical responses. The future may also see the emergence of domain-specific models—such as LLMs trained exclusively on pediatric dentistry content, equipped with citation engines and ethical safety nets. These innovations could transform patient education, clinical triage, and academic content generation. Future studies should also include multilingual assessments, longitudinal tracking of model evolution, and real-world simulations using advanced prompt engineering techniques to more authentically replicate clinician interactions.

## Conclusions

ChatGPT-4o consistently produced the most clinically accurate, contextually relevant, and readable responses among the evaluated models. In contrast, DeepSeek-V3 and Claude 3.7 Sonnet demonstrated weaker performance. While Claude 3.5 Haiku achieved the highest readability scores, it lacked the clinical depth necessary for reliable use in pediatric dentistry. The observed variation in originality and readability metrics highlights the importance of cautious and selective integration of large language models into clinical workflows.

## Figures and Tables

**Table 1:** Summary of the LLMs used in the present study.

Model	Developer	Free Version Model	Paid Version Model	Primary Purpose / Intended Tasks
ChatGPT	OpenAI	GPT-3.5	GPT-4o	Created by OpenAI, ChatGPT is a multimodal AI model supporting writing, tutoring, and coding, widely used for research, education, and problem-solving across disciplines.
Claude	Anthropic	Claude 3.7 Sonnet	Claude 3.5 Haiku	Built by Anthropic, Claude offers safe, honest AI support for writing, analysis, and research, prioritizing ethical use, factual accuracy, and user adaptability across academic and professional domains.
DeepSeek	DeepSeek AI	DeepSeek-V3	N.A.	DeepSeek is a free, multilingual AI assistant for writing, coding, and document analysis, emphasizing accessibility, high-context capacity, and educational support without financial restrictions.
Grok	xAI	Grok 3 mini	Grok 3	Developed by xAI, Grok delivers science-oriented, real-time responses using contextual data, promoting deep understanding and clarity across complex queries and exploratory tasks.
Gemini	Google	Gemini 2.0	Gemini 2.5	Google's Gemini handles multimodal tasks across text, code, and media, offering intelligent content generation, translation, and analysis with high conversational fluency and productivity support.

(AI - Artificial Intelligence)

**Table 2:** Modified Global Quality Scale used in the present study.

Modified Global Quality Scale	
Score 1	Poor quality, poor flow of the information, most information missing, not at all useful for patients or education
Score 2	Generally poor quality and flow, some information listed but many important topics missing, of very limited use to patients or education
Score 3	Moderate quality, suboptimal flow, some important information adequately discussed but others poorly discussed, somewhat useful for patients or education
Score 4	Good quality and generally good flow. Most of the relevant information is listed but some topics are not listed. useful for patients or education
Score 5	Excellent quality and flow, very useful for patients or education

**Table 3:** Demographics characteristics of the pediatric dentists who evaluated the responses of LLMs.

Patient Demographic details		
Gender [n(%)]	Male	5 (50%)
	Female	5 (50%)
Designation [n(%)]	Clinician	0 (0%)
	Academician	5 (50%)
	Both	5 (50%)
Age (mean ± SD)	33.8 ± 2.04	
Years of Experience (mean ± SD)	8.7 ± 1.49	
AI Experience (mean ± SD)	0.8 ± 0.42	

**Table 4:** Comparison of MGQS scores between the LLMs used in the present study.

MGQS Score	Mean ± SD	Median	One way ANOVA	p-value
ChatGPT-3.5	3.45 ± 0.27	3.45	130.94	0.001
ChatGPT-4o	4.28 ± 0.24	4.30		
DeepSeek-V3	2.18 ± 0.19	2.15		
Gemini 2.5	3.33 ± 0.30	3.30		
Gemini 2.0	3.18 ± 0.28	3.20		
Grok-3	3.02 ± 0.19	3.01		
Grok-3 mini	2.79 ± 0.16	2.80		
Claude 3.5 Haiku	2.77 ± 0.20	2.75		
Claude 3.7 Sonnet	2.60 ± 0.24	2.60		

**Table 5:** Comparison of MGQS scores between LLMs according to topic of the questions.

MGQS Score mean	ChatGPT-3.5	ChatGPT-4o	DeepSeek-V3	Grok-3	Grok-3 mini	Claude 3.7 Sonnet	Claude 3.5 Haiku	Gemini 2.5	Gemini 2.0	One way ANOVA value	P value
Growth (N-18)	3.45	4.25	2.2	3.2	2.9	2.8	2.85	3.15	3.15	27.02	0.001
Trauma (N-54)	3.40	4.32	2.12	2.93	2.72	2.43	2.67	3.28	3.22	72.62	0.001
Dental caries (N-27)	3.43	4.23	2.17	3.00	2.70	2.57	2.77	3.33	3.17	10.45	0.001
Oral hygiene (N-45)	3.56	4.18	2.28	3.12	2.94	2.72	2.88	3.48	3.28	19.96	0.001
Habits (N-18)	3.45	4.35	2.25	3	2.8	2.75	2.8	3.35	3.05	13.32	0.001
Drug (N-18)	3.30	4.45	2.05	2.85	2.65	2.45	2.65	3.20	3.00	10.24	0.001

**Table 6:** Comparison of Flesch reading ease scores between the LLMs used in the present study.

Flesch Reading Ease Score	Mean ± SD	One way ANOVA	p-value
ChatGPT-3.5	65.65 ± 5.14	4.73	0.001
ChatGPT-4o	66.24 ± 6.95		
DeepSeek-V3	67.61 ± 6.48		
Gemini 2.5	65.77 ± 3.01		
Gemini 2.0	62.42 ± 7.40		
Grok-3	66.94 ± 4.05		
Grok-3 mini	64.54 ± 4.03		
Claude 3.5 Haiku	70.72 ± 4.06		
Claude 3.7 Sonnet	62.90 ± 3.16		

**Table 7:** Comparison of Flesch Kincaid grade level scores between the LLMs used in the present study.

Flesch-Kincaid Grade Level Score	Mean ± SD	One way ANOVA	p-value
ChatGPT-3.5	9.10 ± 1.60	2.34	0.044
ChatGPT-4o	9.40 ± 2.48		
DeepSeek-V3	8.66 ± 1.73		
Gemini 2.5	7.42 ± 0.96		
Gemini 2.0	15.25 ± 22.92		
Grok-3	9.02 ± 1.33		
Grok-3 mini	9.24 ± 1.67		
Claude 3.5 Haiku	6.82 ± 0.90		
Claude 3.7 Sonnet	9.11 ± 1.01		

**Table 8:** Comparison of Similarity index between the LLMs used in the present study.

	Similarity Index	One way ANOVA	p-value
ChatGPT-3.5	6%	1.43	0.34
ChatGPT-4o	3%		
DeepSeek-V3	3%		
Gemini 2.5	6%		
Gemini 2.0	10%		
Grok-3	13%		
Grok-3 mini	2%		
Claude 3.5 Haiku	1%		
Claude 3.7 Sonnet	9%		

## Data Availability

The datasets used and/or analyzed during the current study are available from the corresponding author.
